# Religious education can contribute to adolescent mental health in school settings

**DOI:** 10.1186/s13033-019-0286-7

**Published:** 2019-04-26

**Authors:** Crystal Amiel M. Estrada, Marian Fe Theresa C. Lomboy, Ernesto R. Gregorio, Emmy Amalia, Cynthia R. Leynes, Romeo R. Quizon, Jun Kobayashi

**Affiliations:** 1 Department of Global Health, School of Health Sciences, University of the Ryukyus, Okinawa, Japan; 2 Department of Environmental and Occupational Health, College of Public Health, University of the Philippines Manila, Manila, Philippines; 3 Department of Health Promotion and Education, College of Public Health, University of the Philippines Manila, Manila, Philippines; 4 Faculty of Medicine, Mataram University, Mataram, Indonesia; 5 Department of Psychiatry and Behavioral Medicine, College of Medicine, University of the Philippines Manila, Manila, Philippines; 6 Japanese Consortium for Global School Health Research, Nishihara, Japan

**Keywords:** Religious education, Adolescent mental health, Schools

## Abstract

**Background:**

Mental disorders contribute substantial burden to the society due to their widespread occurrence and debilitating effects. A quarter of the world’s population are children and adolescents, a significant number of whom experience mental disorders as early as the age of 14. Some interventions have been found to effectively reduce the risk factors and reinforce protective or preventive factors. However, there is still a need to put emphasis on mental health promotion strategies such as religious education. This paper aims to discuss the importance of religious education in promoting mental health.

**Discussion:**

Religious education can be instrumental to improving adolescent mental health. Specifically, it can: (1) help develop healthier reaction to stimuli through the internalization of religious morality; (2) reinforce religious coping mechanisms which reduce the impact of stresses, enhance coping skills, and promote a less risky lifestyle; (3) increase awareness regarding religious beliefs and practices and their influence on the individual, the family, and the community; and finally, (4) promote connectedness which can enhance self-esteem and well-being. However, negative health outcomes such as discrimination and social isolation can also develop, especially among religious or gender minority groups.

**Conclusion:**

It is important to reflect on the crucial role of religious education on adolescent mental health. School-based mental health education and promotion strategies can maximize the benefits of religious education by putting emphasis on effective implementation of religious education to positively influence adolescent mental health.

## Background

Mental disorders are global public health concerns due to their widespread occurrences and debilitating effects. In 2010, mental, neurological, and substance abuse disorders accounted for 258 million disability-adjusted life years (DALYs), equivalent to 10.4% of global DALYs, 2.3% of global years of life lost (YLLs), and 28.5% of global years lived with disability (YLDs) [1]. The most recent estimates from the World Health Organization identified depressive disorders as the largest contributor to global YLD, accounting for 50 million or 7.5% of all YLDs [2].

Adolescence is a critical period of cognitive and behavioral human development. According to Erik Erikson’s Social-Emotional Development Theory, it is during this stage when an individual urgently needs to search for a proper role model to answer the big question of who he/she is and his/her moral and spiritual aspects. This formation of identity is a major event in the development of personality and is associated with positive life outcomes [3]. Moreover, it is during this period when an individual develops the capacity to understand and internalize religion—its beliefs, values, and practices [4], which can lead to changes in the pattern of religious participation [5]. Furthermore, the emergence of mental disorders coincides with this developmental period, with an estimated half of mental disorders manifesting as early as 14 years old [6]. On a global scale, 10–20% of this population experience mental disorders, with depression contributing the largest burden of disease [3]. Poor mental health has been shown to affect the overall health and well-being of adolescents and is associated with adverse health and social outcomes like substance abuse, adolescent pregnancy, school drop-out, and delinquent behaviors, among others.

Mental disorders contribute substantial burden to the society. Given the magnitude of this public health problem, it is necessary to employ strategies which can effectively reduce its occurrence. Strategies which put emphasis on improving social determinants of health such as nutrition, housing, access to education, reductions in economic insecurity and harm from addictive substances, and strengthening of community networks have been found to reduce risks, reinforce protective factors, and decrease psychiatric symptoms and disability and the onset of some mental disorders [7]. School-based interventions are especially advantageous in addressing mental disorders, since children and adolescents spend more time in this setting than in any other formal institutions. Interventions implemented in schools which aim to improve adolescent mental health using strategies such as gatekeeper training and improving knowledge or mental health literacy among others are found to be effective in increasing knowledge, self-esteem, and social support thus reducing risk factors [8–10]; however, there is limited information on the integration of religious education in these interventions, which can be an effective approach in promoting mental health. This paper discusses religious education and how it can influence adolescent mental health.

## Discussion

### Defining religious education

Religious education aims to provide students with knowledge and understanding of, as well as to develop sensitivity to different religions [11]. Religious education has been traditionally categorized into: (a) *confessional religious education*, which seeks to promote obligation towards a specific religion, such as Islam [12], or Catholicism [13]; and (b) *non*-*confessional religious education*, which focuses on providing information about religion/religions for students to expand their understanding on the different worldviews and eventually result in the development of tolerance for other religions [14]. Confessional religious education is implemented in countries such Austria and Croatia, while non-confessional religious education is present in Slovenia [15]. Education is crucial to the development of a tolerant society. The education system in many countries have evolved from being secular to one that promotes pluralism, an understanding that people will have different conceptions of the good and understanding of the best way to live life morally [16]. In the United States, for example, public education was designed to teach Protestant values. However, the increase in the Catholic population has led to the instruction of religion as a subject matter without any intention of indoctrination. Religious education remains a topic for debate because of the inviolable separation of the Church and State in some countries [17]. Nevertheless, teaching religion in secular schools can provide a student with deeper understanding of different cultures around the world, enrich a student’s understanding of human experiences, and allow the student to acquire values that they can integrate into their own lives [18].

### Adolescence and religion

Adolescence is the stage of human development which Erik Erikson states as a transition period from childhood to adulthood. An integral component of this developmental stage is identity development, wherein an individual develops the ability to think about abstract concepts and the capacity to think about the consequences of decisions that they make. This developmental stage is also characterized by an increase in their sense of autonomy, leading to more interaction with peers and other individuals [3]. These changes that occur in adolescents affect their view about religion and its accompanying beliefs and practices. In their quest to develop their identity, they start searching for life’s meaning and become critical of ideologies being taught in religion. Consequently, adolescents question or reject some religious ideas which were taught to them by adults during early age [19]. Experiences and interaction with others during this developmental period are also critical in the development of religiosity. For example, family structure and attachment influence how religious behaviors and attitudes are transmitted from parents to their children; adolescents who were raised by both parents and grew up in families with close relationships are more likely to adopt their parents’ religious beliefs and practices [20]. Similarly, peers influence adolescent religiosity in the sense that religiosity fosters greater peer attachment; that is, religious adolescents tend to have more friends who foster the same religious belief and they are less likely to engage in delinquent behavior [21].

### The role of religious education in adolescent mental health

Religion and its effect on health and well-being has been the subject of many previous studies [22]. Although the definitions for both religiosity and spirituality remains a point of debate among researchers, the two concepts are considered as related [23] and include several dimensions such as beliefs, attitudes, and behaviors, to name a few [24]. A recent systematic review and meta-analysis of randomized controlled trials which investigated the effects of religious and spiritual interventions showed positive effects on mental health outcomes such as significant decrease in stress, alcoholism, and depression [25]. However, the importance of religious education in the promotion of both physical and mental health in the school has not been given emphasis despite several religions teaching about overall health and well-being. For example, Islam teaches the importance of personal hygiene, stress management, and eating healthy [26]; Buddhism teaches avoidance of any drink or drug which can cloud the mind [27]; the Catholic Christian spiritual perspective puts emphasis on confession of sins for forgiveness by God to provide relief to a troubled mind; and Jewish beliefs emphasize that their bodies belong to God and should therefore take care of it by maintaining a proper diet, getting adequate exercise and sleep, maintaining good hygiene, and having a healthy mind [28].

One of the basic aims of religious education is to promote awareness about religious beliefs and practices and how these affect the individual, the family, and the community [29]. Previous studies have presented evidence of the positive effects of religious education against risky health behaviors such as alcohol use, drug use, violence [30], and suicidal ideation [31]. Religious education can be instrumental to improving adolescent mental health by developing religious morality, reinforcing religious coping, developing respect for religious diversity, and promoting connectedness.

Religious beliefs and practices contribute substantially to the development of personal morality and sound judgment, which influence decisions that shape one’s life. Religious education strengthens the formation of moral consciousness through the internalization of religious morality [32]. Internalization, the process wherein an individual adopts the values or regulations prescribed by a religion as their own [33], can affect mental health [34]. Religious moral beliefs such as objections to suicide can also influence suicide rates and attitudes towards suicide [35].

Religious beliefs also affect how individuals deal with stressful situations, suffering, and life problems [36] as it enhances acceptance and one’s ability to function competently in the face of stress and adversity [37]. Religious education reinforces religious coping, which is the use of cognitive behavioral techniques to manage stressful situations in light of one’s spirituality or religious beliefs [38]. Previous studies have shown that people frequently count on religion to cope with stressful situations [35, 36, 39, 40]. Furthermore, meta-analyses have already indicated the positive impacts of religious coping on health wellbeing [41–43]. Positive religious coping involves engaging in religious practices, seeking social support through religious leaders and congregations, and reframing stressful events in reference to their relationship with God [43–45]. Some of these practices and religious involvement have been proven to help in dealing with stressful situations, anxiety, and isolation, displacement after natural disasters, among others [46]. For example, meditation and yoga have been demonstrated to relieve tension and anxiety and stabilize emotions. Traditionally, studies have examined religious coping among Christian populations. However, ethnic minorities living as long-term residents in Western countries have begun to receive attention. These findings suggest that disempowered and deprived groups, including women and ethnic minorities, resort to the use of religious coping and found great efficacy in it [43]. In addition, religious coping has been shown to be extensively used when situations are deemed uncontrollable [41, 47], thus providing an outlet for those at a social disadvantage and with limited access to external resources [48]. Based on these findings, it is apparent that religious coping serves as a resilience mechanism as it enhances a more stable and positive measures of wellbeing. The use of religious coping can enhance contentment with one’s life, thus, potentially protecting against the long-term effects of distress [49].

Religious education can likewise serve as a vehicle to emphasize respect for diversity, by providing a venue for adolescents to understand the differences in religions and world views. Social skills are needed to live and work together harmoniously and to function effectively in a diverse society [50]. It is likewise vital to developing good relationships and values and respecting differences at a personal level [51]. By talking about other’s beliefs and traditions, students are equipped with social skills and the ability to prevent prejudice and hatred towards others [52]. Exploring issues within and across faiths can teach children and adolescents how to understand and respect different religions, beliefs, values and traditions (including ethical life stances), and their influence on individuals, societies, communities and cultures [53]. Putting emphasis on religious diversity can eventually reduce, if not eliminate, cases of bullying, offensive behavior, and violence [54].

Schools exist not only to provide academic knowledge to students, but also to promote connectedness among its students and teachers. Through religious education, the learning atmosphere can be improved by connecting students regardless of their faith [50]. It has been found that receiving religious education provided students with a higher purpose in life and more social support from peers [55]. It can also be a means for teaching religious beliefs and practices which promote support and care for one another. The social support one receives from being part of religious communities and participating in religious practices contributes to positive mental health and well-being [56, 57]. This social support, which accounts largely to the impact of religiosity to well-being, can be credited to the concept of love and brotherhood being promoted by religious teachings [58]. Research has shown that participating in religious practices has positive effects on adolescents in terms of having higher self-esteem and lower incidence of substance abuse through engaging in religious practices, adolescents achieved a feeling of belongingness [22]. Furthermore, social relationships formed through religious involvement are likewise found to intervene with adolescent suicide ideation [59].

Previous studies have also reported some negative effects of religion and religious education on mental health. In some cases, religious beliefs and practices contributed to the development of certain disorders like obsession, anxiety, and depression [60]. In other cases, religiosity was cited as a deterrent in seeking professional help for mental health [61–63]. There are also conflicting findings regarding radicalism and extremism due to religious education. In the case of Pakistan and Indonesia, religious educational institutions were reported to be exploited by perpetrators of extremism [64, 65]. However, others argue that there is no sufficient evidence to conclude that Islamic education contributes to extremism; on the contrary it is reported to foster civic empowerment among students [66].

Additionally, students who are considered as religious minority are also at risk to developing negative mental health outcomes. For example, non-Christian students attending a school where majority of the population are Christians can experience religious discrimination or microaggressions [67]. A previous study has also found that a religious “mismatch” (i.e. students attending a school with religious education which is different from their religion) increased the risk of suicide attempts and self-harm significantly [68]. Other issues identified by previous studies include concerns about schools as being safe spaces [69].

Aside from negative effects experienced by religious minority, religious education can also negatively impact adolescents who belong to sexual minority groups. As discussed previously, adolescence is an important period of personal development and sexual identity development figures prominently during this stage. However, previous studies have reported negative effects of religious education on lesbian, gay, bisexual, transgender, and queer (or questioning) (LGBTQ) adolescents. Although gender discrimination is not unique to religious schools, hostile messages promoted by religious denominations and groups can foster victimization of LGBTQ adolescents [70–72]. These religiously-based messages of discrimination can contribute to social exclusion. Furthermore, non-acceptance of LGBTQ views (i.e. not accepted or permitted in school work) can also undermine academic achievement [72]. Despite, these unintended negative outcomes, religion remains an important aspect of human life, and if implemented properly, religious education can positively influence adolescent mental health.

## Conclusion

Schools are an effective setting for gathering large populations of adolescents for mental health promotion and it is also important to reflect on the crucial role of religious education on mental health among this age group. School-based mental health education and promotion strategies can maximize the benefits of religious education by putting emphasis on effective implementation of religious education to influence adolescent mental health.

## Abbreviations

DALY: disability-adjusted life years
LGBTQ: lesbian, gay, bisexual, transgender, and queer (or questioning)
YLD: years of life lived with disability
YLL: years of life lost
